# Osteogenic Induction of Wharton's Jelly-Derived Mesenchymal Stem Cell for Bone Regeneration: A Systematic Review

**DOI:** 10.1155/2018/2406462

**Published:** 2018-11-11

**Authors:** Ayu Suraya Ansari, Muhammad Dain Yazid, Nur Qisya Afifah Veronica Sainik, Rabiatul Adawiyah Razali, Aminuddin Bin Saim, Ruszymah Bt Hj Idrus

**Affiliations:** ^1^Department of Physiology, Faculty of Medicine, Universiti Kebangsaan Malaysia Medical Centre, Jalan Yaacob Latif, 56000 Cheras, Kuala Lumpur, Malaysia; ^2^Tissue Engineering Centre, Universiti Kebangsaan Malaysia Medical Centre, Jalan Yaacob Latif, 56000 Cheras, Kuala Lumpur, Malaysia; ^3^Ear, Nose & Throat Consultant Clinic, Ampang Puteri Specialist Hospital, 68000 Ampang, Selangor, Malaysia

## Abstract

Wharton's jelly-derived mesenchymal stem cells (WJ-MSCs) are emerging as a promising source for bone regeneration in the treatment of bone defects. Previous studies have reported the ability of WJ-MSCs to be induced into the osteogenic lineage. The purpose of this review was to systematically assess the potential of WJ-MSC differentiation into the osteogenic lineage. A comprehensive search was conducted in Medline via Ebscohost and Scopus, where relevant studies published between 1961 and 2018 were selected. The main inclusion criteria were that articles must be primary studies published in English evaluating osteogenic induction of WJ-MSCs. The literature search identified 92 related articles, but only 18 articles met the inclusion criteria. These include two animal studies, three articles containing both *in vitro* and *in vivo* assessments, and 13 articles on *in vitro* studies, all of which are discussed in this review. There were two types of osteogenic induction used in these studies, either chemical or physical. The studies demonstrate that WJ-MSCs are able to differentiate into osteogenic lineage and promote osteogenesis. In light of these observations, it is suggested that WJ-MSCs can be a potential source of stem cells for osteogenic induction, as an alternative to bone marrow-derived mesenchymal stem cells.

## 1. Introduction

### 1.1. Human Umbilical Cord

**Figure 1:**
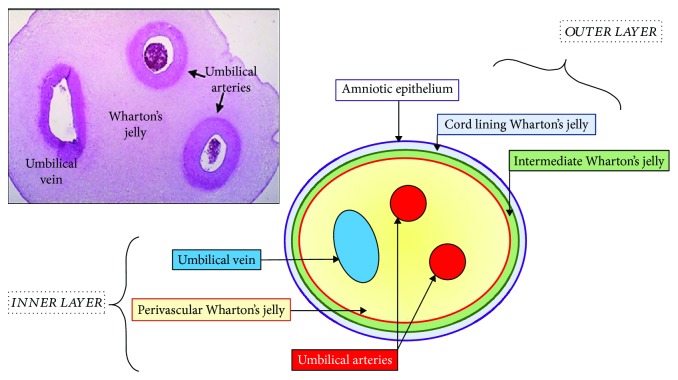
Anatomical compartment of Wharton's jelly mesenchymal stem cell.

About 25 years ago, the umbilical cord was considered to be a type of medical waste, until it was found to be a rich source of stem cells [1]. The abundance of stem cells and the ease of isolation have become deciding factors while choosing the source of adult stem cells. The human umbilical cord (HUC) is approximately 65 cm in length and 1.5 cm in diameter [2, 3]; it connects the fetus to the mother and supplies nourishment. The cord is covered by single or multiple layer of squamous cubic epithelial cells derived from the developing amnion [4–6]. The HUC contains two arteries and one vein, which are surrounded by a mucoid connective tissue known as Wharton's jelly (WJ) [7]. This mucous connective tissue is made up of mucopolysaccharides that are hyaluronic acid and chondroitin sulphate [8]. Basically, WJ can be divided into 4 layers with the outer layer being the amniotic epithelium followed by cord lining WJ and intermediate WJ. The inner layer of WJ is also known as perivascular WJ which contains arteries and vein [7] (Figure 1).

It has been reported that WJ also contains myofibroblast-like stromal cells, collagen fibers, proteoglycans, fibroblasts, and macrophages. WJ has garnered interest due to its availability, the noninvasive method of collection, and high cell yields. It has been demonstrated that mesenchymal stem cells (MSCs) isolated from the umbilical cord express matrix receptors (CD44 and CD105) and integrins (CD29 and CD51), but not hematopoietic lineage markers (CD34 and CD45) [9]. These cells exhibit a phenotype similar to that of mesenchymal stem cells from other tissue sources [10]. According to the International Society for Cellular Therapy (ISCT), stem cells should demonstrate plastic adherence, not expressing hematopoietic markers, and be able to commit to the adipogenic, osteogenic, and chondrogenic lineage [11]. WJ-MSCs have a huge advantage where the phenotype and stemness are remained despite being in a long-term culture. This enables a mass production of cells which is usually required for regenerative medicine [12].

### 1.2. WJ-MSCs and Their Potential in the Treatment of Diseases

WJ, also known as *substantia gelatinea funiculi umbilicalis*, consists of fibroblast-like cells and mast cells that are embedded in proteoglycans, mainly hyaluronic acid. The cells are thought to be trapped in WJ during the early stage of embryogenesis, when they migrate from the aortic gonadotropin mesonephric region to the fetal liver through the umbilical cord [13]. WJ-MSCs have many advantages over other types of stem cells, including higher proliferation rates and broader multipotency. WJ-MSCs are able to differentiate into many cell types such as adipocytes [14, 15], osteoblasts [14, 16], hepatocytes [17], chondrocytes [18], and neural cells [19, 20]. Interestingly, it has also been demonstrated in a three-dimensional model that WJ-MSCs are able to differentiate *in vitro* into cornea epithelial-like cells, which may offer a solution for patients with limbus stem cell deficiency [21].

Cui and colleagues have successfully improved cognitive function in a mouse model of Alzheimer's disease using intravenously delivered WJ-MSCs, which reduced oxidative stress and promoted hippocampal neurogenesis [20]. In a clinical trial conducted by Hu and colleagues, an intravenous infusion of WJ-MSCs in type 2 diabetes mellitus patients improved the function of islet *β*-cells and reduced the incidence of diabetic complications [22]. Additionally, WJ-MSC therapy is now used to treat corneal epithelial, stromal, and endothelial disorders apart from the conventional intervention such as surgery, ionizing radiation, or drug treatment [23, 24].

### 1.3. Immunomodulatory Aspect of WJ-MSCs and BM-MSCs

WJ harbours MSCs that possess a similar phenotype as harvested from the bone marrow and other sources. WJ-MSCs do not express HLA-DR and costimulatory molecules CD40, CD80, and CD86 which are essential for the activation of T-cells [1, 2, 9, 25–27]. WJ-MSCs have been proven to have lower immunogenicity than BM-MSCs as depicted by Weiss et al. [1] who conducted an experiment using mixed lymphocyte reaction (MCR) assay [26]. In a study conducted by Prasanna et al. and Deuse et al., both consistently showed low HLA-DR expression compared to BM-MSCs after stimulation with IFN-*γ* and proinflammatory cytokines [28, 29]. In normal culture conditions, HLA-DR is not expressed; hence, the activation of T-cell is inhibited, reducing the risk of allograft rejection which potentially makes it safe for human transplantation.

It is documented that BM-MSCs harbour viruses, which is a major drawback in clinical application. There are reports from patients who undergo BM-MSC transplant who are infected with viral infection as a complication of the cell-based therapy [9]. Interestingly, the virus can escape from detection therefore increasing the morbidity and mortality [30]. Moreover, there are various diseases such as aplastic anemia, leukaemia, and bone marrow failure that impedes the application of BM-MSCs in therapy [31].

### 1.4. WJ-MSC Homing and Migration for Bone Healing

Generally, MSCs are known to migrate towards the injury site and help the healing process. The migration process, known as homing, is defined as the arrest of MSCs within the vasculature of a tissue before it crosses over the endothelium [24, 32]. As for now, the mechanism of MSC homing is still vague and BM-MSCs are postulated to have a mechanism similar to leukocyte homing. The mechanism is initiated when MSCs collide with the endothelium rolling, causing a slackening of cells in the blood flow. The G-protein-coupled receptors activated the cells and activated integrin mediation, causing activation-dependent arrest. The process is completed with the transmigration of the cells through the endothelium and the underlying basement membrane [33]. There are several factors that contribute to the homing mechanism which are growth factor expression as well as chemokine and extracellular matrix receptors on the MSCs' surfaces [34].

In a study conducted by Granero-Moltó et al., MSCs are migrated to the fracture site via the CXCR4 receptor causing improvement of biomechanical properties and increasing the cartilage and bone of the callus [24]. Zwingenberger et al. demonstrated that the combination of the SDF-1 released and bone morphogenetic protein 2 contributes to the migration of the stem cell [35]. This occurrence markedly boosts bone regeneration. Apart from that, MSCs are also recruited towards wear-particle-related osteolysis which is indicated by the inflammatory macrophage that is also the chemokine CC receptor (CCR) 1 of MSCs [30, 36]. The MSCs are demonstrated to increase bone mineral density and decrease the osteolytic process [37].

### 1.5. Cellular Mechanism of Bone Remodelling for Bone Regeneration

Bone repair or bone regeneration is characterized by a series of tissue transformation mechanisms including resorption and formation of hard and soft tissue. Therefore, mineralized tissue remodelling is required for the involvement of various cell types including osteoclast and osteoblast. Osteoblasts are bone-forming cells that can be found at the surface of bone, while osteoclasts are multinucleated bone-resorbing cells derived from bone marrow stem cells [38]. Bone remodelling is a cyclical process in which bone undergoes consistent renewal to ensure the replacement of primary bone, to maintain calcium homeostasis, and especially to heal ischemic and microfractured bone [7, 20, 32–34, 39]. This process requires a correct balance of bone resorption and bone formation and thus involves osteoclasts and osteoblasts, respectively.

**Figure 2:**
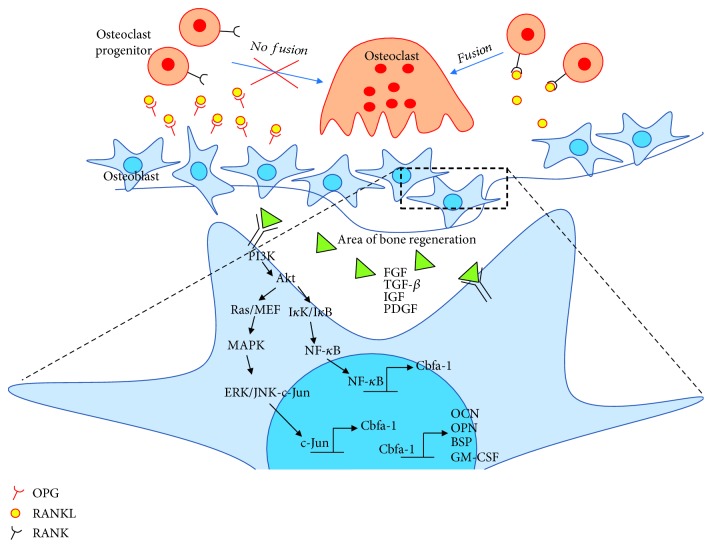
Mechanism of bone regeneration and activation of signaling pathways.

Bone remodelling consists of five consecutive phases: (1) the resorption phase, where osteoclasts break down the bone tissue, resulting in mineral release; (2) the reversal phase, where mononuclear cells appear on the bone surface; (3) the formation phase, where osteoblasts trapped in the bone matrix become osteocytes; (4) the mineralization phase, where osteocytes produce type I collagen and other substances that make up the bone extracellular matrix; and (5) the termination phase [38, 40]. Resorption is initiated by osteoclast progenitors that are recruited and disseminated into the bloodstream. These cells proliferate and differentiate into mature osteoclasts, aided by osteoblast stromal cells via cell-to-cell interactions. These osteoblasts express two cytokines, i.e., receptor activator of NF-*κ*B ligand (RANKL) and osteoprotegrin (OPG), involved in osteoclast progenitor cell differentiation. Under parathyroid hormone (PTH) stimulation, RANKL will bind to RANK, a cytoplasmic membrane receptor on osteoclast progenitor cells, to stimulate their fusion, differentiation, and activation. In contrast, OPG binds to RANKL to counterbalance the effect of RANKL-RANK, which thereby determines the extent of bone resorption. These events are important in maintaining bone homeostasis [41] (Figure 2). Bone resorption is terminated when osteoclasts undergo apoptosis and the reversal phase is initiated. Reversal cells may thus represent the missing link necessary to understand the coupling between bone resorption and formation. Researchers have found that reversal cells colonizing the resorbed bone surface are immature osteoblastic cells that gradually mature into bone-forming osteoblasts during the reversal phase and prepare the bone surface for bone formation [42].

### 1.6. Molecular Mechanism of Bone Remodelling for Bone Regeneration

The differentiation of MSCs depends on which signalling pathway is activated. Apart from osteoblasts, WJ-MSCs have also been demonstrated to differentiate into other mesenchymal cell lineages such as hepatocytes [43], chondrocytes [18, 44], and adipocytes [14, 15]. The markers for osteogenic differentiation are alkaline phosphatase (ALP), an early marker of osteogenic differentiation and mineralization, and RUNX2, a runt domain-containing transcription factor that is crucial for osteogenic differentiation and bone formation. The activation of RUNX2 triggers COL1 (collagen type 1), osteopontin (OPN), and osteocalcin (OC), which are osteoblast-specific markers. OPN is expressed later in the differentiation stage [45, 46]. Both OPN and COL1 are synthesized by osteoblasts. OC is also expressed later and is important for maintaining bone resorption. Osterix (Osx) is a downstream factor of Runx2 that binds to activated NFAT2 in bone development [47]. A study by Zhou et al. explored the function of Osx where it regulates bone homeostasis after birth for bone and cartilage formation [26].

In osteoblasts, lineage-specific gene expression control by specific transcription factors, i.e., Cbfa-1/RUNX2, acts to regulate osteoblastic specific gene expression [48]. Cbfa-1/RUNX2 is required for osteoblast differentiation, since Cbfa-1 knockout mice display impaired or even absent bone formation [49, 50]. This transcription factor contains a runt DNA-binding domain, which can bind to DNA as a monomer or as a subunit of a monomeric complex. It binds to various enhancers and promoters, including those for the genes encoding osteocalcin, osteopontin, bone sialoprotein, and GM-CSF. The expression of these proteins contributes to the bone matrix, leading to the maturation of osteoblasts. These genes can also be used as markers for different stages of osteoblast development [51–53].

The expression of transcription factors is controlled by several pathways that are activated by growth factors (GFs) that bind to a specific receptor. These growth factors include fibroblast growth factor (FGF), transforming growth factor-*β* (TGF-*β*), insulin-like growth factor (IGF), platelet-derived growth factor (PDGF), and vascular endothelial growth factor (VEGF) [54]. It has been reported that these GFs are responsible for regulating the expression of Cbfa-1/RUNX2 via the MAPK [55], ERK [52], and PI3K-Akt pathways [54, 56]. GF binding to its receptor tyrosine kinase (RTK) activates a downstream signaling cascade. The activated RTK activates class I phosphatidylinositol 3-kinase (PI3K) or guanosine nucleotide-binding protein (Ras) and propagates the signal through direct binding or tyrosine phosphorylation. This then activates Akt/PKB, I*κ*K/I*κ*B, or Raf/MEK, which then activates NF-*κ*B or MAPK, accordingly. Activated NF-*κ*B and MAPK act through direct binding to phosphorylate ERK/JNK-cJun, which then activate Cbfa1/RUNX2 gene expression. TGF-*β* plays important roles in osteoblast precursor recruitment, FGF enhances osteoblast recruitment and proliferation, IGF is involved in the regulation of bone matrix synthesis and migration, VEGF regulates osteoblast differentiation, and PDGF is involved in osteoprogenitor migration [51, 52]. Cumulatively, the osteogenic differentiation capability owned by WJ-MSCs supplemented with specific GF is postulated to have a high potential for bone regeneration.

### 1.7. WJ-MSCs for Bone Regeneration

Albeit many have used WJ-MSCs in the studies, the safety and efficacy of its application are indecisive particularly in bone regeneration. There are several aspects that need to be considered prior to using WJ-MSCs that may influence the yield and its stemness potency. Different parts of the umbilical cord generated diverse frequencies of MSCs and cell populations [6–8, 57–59]. It is known that Runx2 plays a pivotal role in osteoblast differentiation. In a previous study, it has shown that WJ-MSCs have lower capability to differentiate due to the high level of RUNX2 but lower ALP expression. ALP is important for matrix maturation [60, 61]. WJ-MSCs also exhibited higher expression of pluripotent markers, OCT 4, SOX 2, and NANOG than in other parts of the umbilical cord [62]. From those findings, it can be postulated that regulation of Runx2 and pluripotent impedes ALP expression, thus needing a specific modulator that can serve as a molecular switch of WJ-MSCs' fate. Current study by Bustos and colleagues have demonstrated that JARID1B (Jumonji AT-rich interactive domain 1B) histone demethylase represses Runx2 in undifferentiated WJ-MSCs. In JARID1B knockdown murine, it can be seen that Runx2 is highly expressed and ready for osteogenic commitment indicating that this molecular mechanism is relevant to modulating osteoblastic lineage commitment [63].

### 1.8. Clinical Application of WJ-MSCs in Bone Regeneration

The current standard commonly used for bone tissue replacement is bone grafting obtained from patients themselves (autograft) or from other individuals (allograft). However, this has raised various effects including immunoreactivity and infection as well as procedure. WJ-MSCs have proved its capability to help in bone regeneration for clinical application. Qu et al. treated 36 patients with nonunion bone fracture with WJ-MSCs cultured with platelet-rich plasma (PRP) resulting in a faster recovery with no infection recorded compared to the other 36 patients with autoiliac treatment [64]. In another study, the intravenous injection of 3–5 million WJ-MSCs alleviated the condition of Becker muscular dystrophy patients with increased muscle strength, improved appetite, and also improved patient's gait [65]. Recently, a patient in Indonesia with infected nonunion bone is able to walk with no pain and no postoperative complications recorded after local implantation of 5 million cells supplemented with BMP-2 and hydroxyapatite [66]. A transplantation of autologous BM-MSCs and allogeneic WJ-MSCs in treating osteonecrosis of femoral head showed improvement where it relieved the pain and improved the joint function [67].

To this date, there are many various positive outcomes upon WJ-MSC treatment in clinical trials for different disorders including neurology [67], hematology [68], liver diseases [69], and particularly musculoskeletal diseases [65, 70]. WJ-MSCs have a huge potential to be used as an alternative treatment for bone disorders as it does not require ethical issues to obtain and it is alleviating patients' morbidities [71]. Since WJ-MSCs can retain the stemness and phenotypic stability compared to BM-MSCs, it has potency to be commercialised [72].

## 2. Methods

### 2.1. Search Strategy

A systematic review was conducted to systematically assess articles on the potential of WJ-MSCs for bone regeneration. Two databases were comprehensively used to search for relevant studies, i.e., Medline via Ebscohost and Scopus. For search term keywords, the combination of words used was “Wharton's jelly” AND “osteo^∗^” OR “bone”.

### 2.2. Selection Criteria

The year limit for searches was from 1961 to 2018, and only studies published in English were considered. The search outcomes identified all articles containing the words Wharton's jelly, umbilical cord, osteogenesis, osteogenic, and bone. Databases were searched individually to ensure all relevant studies were considered. The titles and abstracts were carefully screened for eligibility related to the topic of interest. Primary studies related to bone formation or bone regeneration were included. Review articles, news articles, letters, editorials, and case studies were excluded from the search.

### 2.3. Data Extraction and Management

Data were extracted from each eligible article by two reviewers. The selected papers were screened in several phases prior to inclusion. First, the titles that were not relevant to the topic were excluded. Next, the abstracts of the papers were screened, and unrelated studies were excluded. All duplicates were removed. The following data were summarized from the selected studies: (1) authors, (2) type of study, (3) subject/sample, (4) induction factor, (5) methodology, (6) results, and (7) conclusions.

## 3. Results

### 3.1. Search Results

**Figure 3:**
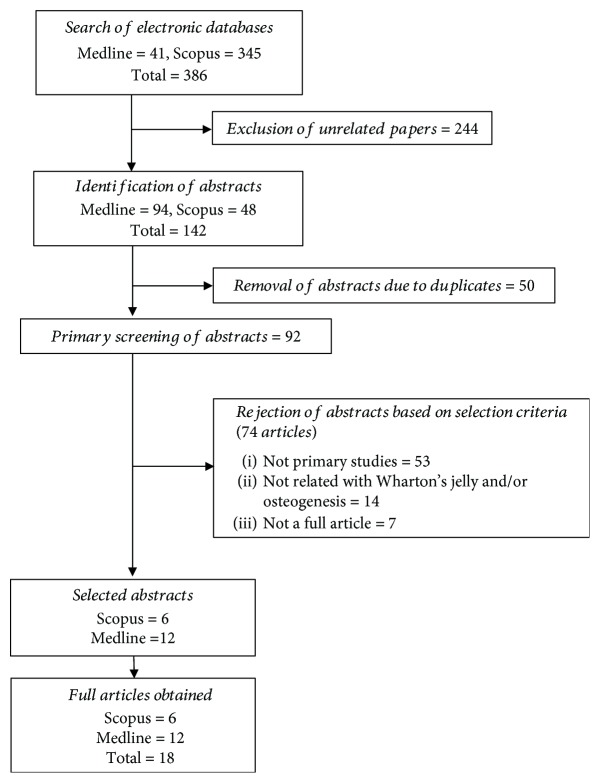
Flow chart of the article selection process using the Scopus and MEDLINE databases.

The primary searches identified 386 articles: 41 articles came from Medline and 345 articles were found in Scopus. To minimize bias and improve the strength of the related articles, two reviewers independently assessed the articles according to the inclusion and exclusion criteria. There were 244 articles removed as they were unrelated to either Wharton's jelly or osteogenesis/bone. A joint discussion was conducted to achieve consensus where differences emerged during the assessment. From the 142 remaining articles, 50 duplicates were removed before full articles were retrieved. From 92 articles, 74 articles were rejected based on the inclusion criteria as the articles were not primary studies, were not related to Wharton's jelly or osteogenesis, or were not available as full articles. Finally, a total of 18 studies were selected for data extraction in this review. The flow chart of the selection process is shown in Figure 3.

### 3.2. Study Characteristics

All studies were published between 1961 and 2018. An article reported on animal studies (in vivo), two articles on both *in vitro* and *in vivo* assessments, and 14 articles on *in vitro* studies. There were six articles which included scaffold fabrication in the study. Three out of six scaffold studies involved the optimization of the scaffold component for better bone regeneration. Eleven studies proposed a different type of tissue as the MSC source. From the generated data, we classified the articles into two subgroups: (1) chemical methods to promote osteogenesis and (2) physical methods (scaffolds) to promote osteogenesis. A summary of the studies is provided in Table 1.

## 4. Discussion

The database search provided 18 articles related to Wharton's jelly, umbilical cord, osteogenesis, osteogenic lineage, and bone. From these articles, various tissue sources were assessed for potential MSCs. Each of these sources was examined regarding MSC differentiation capacities into the adipogenic, chondrogenic, and osteogenic lineages. This review assessed the osteogenic potential of WJ-MSCs, which may have remarkable potential for bone regeneration in the clinic.

### 4.1. Mesenchymal Stem Cells: Potential Sources

Mesenchymal stem cells (MSCs) have attracted attention because of their unique plasticity and ability to differentiate into multiple cell lineages, i.e., osteoblasts, chondrocytes, and adipocytes, with potential for clinical usage. The bone marrow is a primary source of MSCs. However, it has been reported that the frequency as well as the differentiation potential of BM-derived MSCs (BM-MSCs) decline with increasing age [73]. Therefore, alternative sources of MSCs are needed, especially those that can be obtained noninvasively. Currently, various tissues are under consideration for MSC isolation, including adipose tissue, muscle, amniotic fluid, menstrual blood [74, 75], fetal blood [76], and periodontal ligaments (PDL) [61]. The human umbilical cord is a promising source of MSCs, as MSCs can be isolated either from the whole umbilical cord [16, 77], the umbilical vein subendothelium [78], or WJ [14, 19, 74]. One group of researchers divided the umbilical cord into three anatomical segments, i.e., the maternal, middle, and fetal segments [79]. They demonstrated that MSCs from the maternal and fetal segments displayed greater viability, possessed significantly higher proliferation rates, and underwent more complete osteogenic differentiation, showing that these segments are a good source of MSCs for bone tissue engineering [79].

It is important to characterize cells derived from tissues to determine the type of cell population that exists in the preparation. A heterogeneous population could influence the differentiation properties, specifically the osteogenic potential of MSCs for bone regeneration. There are a few surface markers that are commonly reported for MSCs such as CD13, CD29, CD44, CD73, CD90, CD105, and CD166. MSCs do not express CD31, CD144, and CD309 (endothelial cell markers) or CD14, CD34, CD45, CD117, and CD133 (hematopoietic cell markers) [61, 79].

### 4.2. Wharton's Jelly Mesenchymal Stem Cells Undergo Osteogenic Differentiation

WJ-MSCs have been shown to have good potential for osteogenic differentiation. These cells display all features of functional osteocytes/osteoblasts based on osteogenic gene expression, extracellular matrix (ECM) mineralization, and the ability to adhere to a fabricated scaffold [80, 81]. Although WJ-MSCs have been broadly investigated, there are still problems when it comes to transplantation, as an immune response and rejection could occur [82]. In the database search, we only found one article that used autoserum for WJ-MSCs *in vitro* as a substitute for FBS to reduce the rejection rate. Autoserum is serum obtained from the umbilical cord blood. Baba et al. reported that the cell culture medium using autologous serum is superior in quality to medium using FBS. WJ-MSCs cultured in autologous serum exhibited successful osteoblastic and adipogenic differentiation. The WJ-MSCs were then transplanted subcutaneously into nude mice, and their potential to form bone was proven [16].

#### 4.2.1. Chemical Induction

Seven studies out of 18 selected articles used chemical factors to promote osteogenesis in WJ-MSCs. Batsali et al. [83] demonstrated that WJ-MSCs are able to differentiate into the osteogenic lineage, although this was inferior compared to BM-MSCs. They demonstrated that WISP1, a canonical Wnt pathway target protein, was able to promote better osteogenic differentiation in WJ-MSCs [83]. A study by Szepesi et al. [61] showed that adipose tissue-derived mesenchymal stem cells (AT-MSCs) and periodontal ligament-derived mesenchymal stem cells (PDL-MSCs) have excellent potential for bone replacement applications and better endothelial differentiation ability as compared to WJ-MSCs [61]. The high degree of calcification in AT-MSCs and PDL-MSCs demonstrates that calcium deposition was better as compared to WJ-MSCs for generating vascularized bone grafts [61]. Similar findings were reported by [84] where AD-MSCs were found to be superior to WJ-MSCs in terms of differentiating into the osteogenic lineage after 21 days compared. In a different study by Lim and colleagues, MSCs derived from different parts of the umbilical cord, i.e., the fetal, middle, and maternal segments, have the ability to differentiate into osteogenic lineage cells using the osteogenic medium consisting of dexamethasone, ascorbic acid, and *β*-glycerophosphate. The fetal part was shown to have the best differentiation potential [79]. A study by Hsieh et al. [14] showed that BM-MSCs express more osteogenic genes compared to WJ-MSCs; conversely, WJ-MSCs are more responsible for angiogenesis [66]. Bone morphogenetic protein 2 (BMP-2) was used in a study by Hou et al. to promote osteogenic differentiation in WJ-MSCs [15].

#### 4.2.2. Physical Induction

It is noteworthy that the microenvironment influences cell behaviour and leads to the production of a specific chemical composition that builds the ECM. Therefore, fabricated scaffolds have been actively investigated to find better materials and to produce the best structure of ECM-like components. From the database search, eight out of 18 articles investigated the fabrication of various scaffolds to test the potential of WJ-MSCs to promote complex bone regeneration. Various biomaterials were used to construct these scaffolds, ranging from collagen hydrogels [85] to bioactive glass [80]. 3D scaffolds have been documented as one of the best carriers for cell delivery in bone regeneration. The ideal scaffold should be osteoconductive, biocompatible, and bioresorbable; possess interconnected porosity; and promote cell binding/attachment [82, 86].

The first phase in scaffold development used collagen as the main organic component in bone tissue for bone grafting [85]. Collagen type I can be isolated from Sprague-Dawley rat tails after processing and pelleting. Genipin has been selectively used for crosslinking collagen scaffolds to improve the stability and mechanical strength of the scaffolds in the culture medium [74]. Other crosslinkers have also been used, such as glutaraldehyde and formaldehyde. However, those have been reported to have some cytotoxic effects [87]. Calcium phosphate is another major constituent in bone that has been widely studied as a scaffold material for bone tissue engineering [88]. A study by Karadas et al. [74] produced an in situ mineralized collagen scaffold whereby, after crosslinking with genipin, the scaffold was immersed in a calcium and phosphate solution. As a result, highly integrated calcium phosphate minerals were successfully formed [74]. The combination of collagen I and III has also been reported to resemble the native ECM, where umbilical cord MSCs (UC-MSCs) were found to have better osteogenic potential compared to BM-MSCs [85]. In addition, porcine ECM, derived from the urinary bladder, has also been used as a biomaterial for scaffold preparation as it contains collagen, glycoproteins, glycosaminoglycans, and GFs [89].

Scaffold design then moved to the second phase, in which bioactive glass has been used as a scaffold in bone tissue engineering [80]. Kargozar and colleagues used a combination of bioactive glass/gelatin (BaG/Gel) scaffolds, aiming for a highly porous structure, which is considered ideal for bone substitution. A comprehensive physiochemical analysis showed that the structure had an intact, 3D porous microstructure with interconnected pores. It was also shown that the properties were very close to those of natural spongy bone [80]. They demonstrated that neovascularization was significantly better in the UC-MSC-seeded scaffold when compared to the BM-MSC-seeded scaffold, indicating that the BaG/Gel scaffold is MSC type-dependent. A study by Todeschi and colleagues used hydroxyapatite (HA), beta-tricalcium phosphate (*β*-TCP), or a mixture of the two [81] as the scaffold. They showed that a significantly higher number of blood vessels were present in the UC-MSC-seeded implants [81].

## 5. Conclusion

WJ-MSCs were first isolated by Mitchell et al. in 2003. During embryogenesis, totipotent cells such as primordial germ cells and hematopoietic stem cells migrate from the yolk sac through this region to populate target tissues in the embryo and fetus [90]. Characterization indicated that these cells are stem cells, as they express c-kit and can differentiate into neural cells. WJ-MSCs have similar proliferation and differentiation capacity and have multilineage differentiation potential [77], including osteogenesis. This review demonstrates that WJ-MSCs are capable of differentiation into osteoblasts, which may be useful for more effective bone fracture healing as these cells have been shown to migrate into and colonize a collagenous matrix. With the aid of 3D scaffolds, cell proliferation and survival are improved as these scaffolds provide structural stability similar to that of bone. However, MSCs have to be compatible with the scaffold prior to integration and incorporation into engineered bone.

## Figures and Tables

**Table 1:** Summary and classification of the 18 articles selected from the database search.

No.	Author and year	Type of study	Subject/sample	Induction factor	WJ-MSC isolation method	Results	Conclusion
1.	Fu et al. 2018 [91]	*In vitro* and *in vivo*	(i) Human umbilical cord-derived mesenchymal stem cells (UC-MSCs) (ii) Sprague-Dawley's osteoblast (iii) Sprague-Dawley's osteoclast	Differentiation medium Osteogenic medium: (1) High-glucose DMEM (2) 10% serum (3) 10^−8^ M dexamethasone (4) 50 ng/mL L-ascorbic acid (5) 10 mM *β*-glycerophosphate	(i) Enzymatic digestion (ii) Part of UC: not mentioned	(1) MicroCT result showed that transplantation of UC-MSCs increased bone mass in the distal condyle of normal rat femur compared to other groups (2) Goldner's staining indicated that compact arrangement of collagen with less trabecular thickness in the group undergoes implantation with UC-MSCs. A trabeculae-like structure containing lamellae was detected, but there are no cell arrangements found in the area (3) Osteocalcin (OC) staining showed increased osteocalcin level in OVX-receiving UC-MSCs (4) Antihuman-specific nuclei antigen showed engrafted UC-MSCs had differentiated into osteoblasts (5) RT PCR: (i) Human osteocalcin and abundant osterix (OSX) was detected in ovx-receiving UC-MSCs (6) *In vitro* coculture system showed more expression of alkaline phosphatase (ALP) if the osteoblast is cocultured with UC-MSCs	UC-MSCs able to be differentiated into osteoblast and are safe for transplantation in bone disease treatment.
				Osteoclast differentiation (1) 10 or 100 ng RANKL			

2.	Al Jofi et al. 2018 [92]	*In vitro*	(i) Wharton's jelly mesenchymal stem cells (WJ-MSCs)	(i) 10 *μ*mol/L metformin (antidiabetic drug)	(i) Commercial UC-MSCs (ii) Part of UC: not mentioned	Metformin-treated UC-MSCs increased in mineralization stained through Alizarin Red Staining. But in OCT-1- (organic cation transporter-) siRNA-transfected cells, a significant decrease in calcium-rich nodule formation was observed.	OCT-expressing WJ-MSCs have the ability to be differentiated into osteoblasts when induced with metformin.

3.	Bharti et al. (2018) [44]	*In vitro*	(i) Wharton's jelly mesenchymal stem cells (WJ-MSCs)	(i) Osteogenic medium (1) ADMEM (2) 0.1 mM dexamethasone (3) 50 mM ascorbic acid (4) 10 mM glycerol-2-phosphate (ii) Adipogenic medium: (1) ADMEM (2) 500 mM isobutyl methylxanthine (3) 1 mM dexamethasone (4) Insulin (5) 100 mM indomethacin (6) 10 mM insulin (iii) Chondrogenic medium using comer (1) StemPro1Osteocyte/Chondrocyte Differentiation Basal Medium; StemPro1 Chondrogenesis supplement; Gibco). (2) Hepatocyte (3) ADMEM (4) 2% FBS (5) 10 ng/ml of oncostatin M (6) 10 nmol/L dexamethasone (7) 1% insulin transferrin-selenium	(i) Explant method (ii) Part of UC: maternal, middle and fetal segments	(1) Bone nodules: formed by cells from all segments (2) Chondrogenic potential: occurred in cells from all segments	WJ-MSCs are a good cell source for autologous/allogeneic stem cell source.

4.	Batsali et al. 2017 [83]	*In vitro*	(i) Bone marrow mesenchymal stem cell (BM-MSCs) (ii) Wharton's jelly mesenchymal stem cells (WJ-MSCs)	Differentiation medium: Osteogenic medium: (1) High-glucose DMEM (2) 10% serum (3) 10^−7^ M dexamethasone (4) 25 *μ*g/mL L-ascorbic acid (5) 3 mM NaH_2_PO_4_	(i) Explant method (ii) Part of UC: maternal, middle, and fetal segments	(1) Osteogenic differentiation (Alizarin Red and von Kossa staining): WJ-MSCs showed similar staining potential as BM-MSC (2) Real-time- (RT-) PCR: (i) Osteocyte-related gene expression: higher expression of Runt-related transcription factor 2 (RUNX2), distal-less homeobox protein 5 (DLX5), osteocalcin (OCN), and alkaline phosphatase (ALP) by BM-MSCs (3) Differential expression of WNT ligands; sFRP4 (*secreted frizzled-related protein 4 ***)** and WISP1 (WNT1-inducible signalling pathway protein 1) were significantly reduced in WJ-MSCs (4) WISP1 implicated in osteogenic differentiation; RUNX2, ALP, and OSC were significantly upregulated	The osteogenic differentiation potential of WJ-MSCs is regulated by WISP1 and sFRP4, respectively.

5.	Zajdel et al. 2017 [84]	*In vitro*	(i) Adipose tissue (AT-MSCs) (ii) WJ-MSCs	Osteogenic medium (Lonza): (1) Dexamethasone (2) Ascorbic acid (3) *β*-Glycerophosphate	(i) Commercial AT-MSCs and UC-MSCs (ii) Part of UC: not mentioned	(1) Calcium deposition by Alizarin Red staining (i) Calcium deposition was greater in AT-MSCs when compared to WJ-MSCs (2) ALP activity (i) ALP activity was higher in AT-MSCs when compared to WJ-MSCs (3) Osteoprotogerin (OPG) secretion (i) Both osteo-induced cell types showed high OPG secretion when compared to control (4) Osteocalcin (OC) secretion (i) OC secretion was higher in WJ-MSCs when compared to AT-MSCs	WJ-MSCs have the ability to differentiate into the osteogenic lineage.

6.	Mechiche Alami et al. 2017 [88]	*In vitro*	(i) WJ-MSCs	Calcium phosphate (CaP) substrate build-up (without osteogenic induction	(i) Commercial UC-MSCs (ii) Part of UC: not mentioned	(1) Gene expression analysis (i) Day 7: Runt-related transcription factor 2 (RUNX2) and secreted phosphoprotein 1 (SPP-1) were upregulated (ii) Day 14: collagen type 1 Alpha 1 (COL1A1) and ALP were upregulated (iii) Day 21: bone gamma-carboxyglutamic acid-containing protein (BGLP) and ALP were upregulated (2) Nodule characterization (i) Hematoxylin-eosin-saffron (HES) staining revealed continuous layers of cells at the surface of the nodule with randomly distributed cells embedded within fibrous tissues (ii) Masson's trichrome staining showed the presence of green-stained fibrous tissue composed of newly formed collagen	Excellent osteogenic potential of sprayed CaP and WJ-MSCs in bone tissue engineering

7.	Szepesi et al. 2016 [61]	*In vitro*	(i) WJ-MSCs (ii) AT-MSCs (iii) Periodontal ligament MSCs (PDL-MSCs)	Differentiation medium: (i) StemPro Osteogenesis Differentiation kit (ii) StemPro Chondrogenesis Differentiation kit (iii) StemPro Adipogenesis Differentiation kit (iv) Endothelial Cell Growth Medium	(i) Enzymatic digestion UC-MSCs (ii) Part of UC: not mentioned	(1) Osteogenic differentiation: AT-MSCs and PDL-MSCs showed greater calcium deposition (2) Osteogenic differentiation assessed via RT-PCR: (i) Runt-related transcription factor 2 (RUNX2): all cell types showed high expression after induction (ii) Alkaline phosphatase (ALP): AT-MSCs and PDL-MSCs showed increased expression, but ALP was significantly lower in WJ-MSCs (iii) Calcium deposition: AT-MSCs and PDL-MSCs showed greater calcium deposition as compared to WJ-MSCs (3) There was a significant correlation between CD90 expression and the levels of calcium deposition in different MSC isolates	WJ-MSCs have osteogenic potential and are good cell sources for bone regeneration.

8.	Lim et al. 2016 [79]	*In vitro*	Human WJ-MSCs (i) Fetal segment (ii) Maternal segment (iii) Middle segment	(i) Osteogenic medium (1) Alpha-MEM (2) Dexamethasone (3) Ascorbic acid (4) *β*-Glycerol phosphate (ii) Adipogenic medium: (1) DMEM/F12 (2) 3-Isobutyl-3-methylxanthine (3) Dexamethasone (4) Insulin (iii) Chondrogenic medium (1) Alpha-MEM (2) Transforming growth factor 3 (TGF-*β*3)	(i) Enzymatic digestion (ii) Maternal, middle, and fetal segments	Bone nodules: formed by cells from all segments	WJ-MSCs are a good cell source for bone regeneration.

9.	Kargozar et al. 2018 [80]	*In vitro* and *in vivo*	(i) BM-MSCs (ii) AT-MSCs (iii) UC-MSCs	(i) Nanocomposite scaffolds (3D bioactive glass/gelatin scaffolds (BaG/Gel) consisting of SiO_2_-P_2_O_5_-CaO (64% SiO_2_, 5% P_2_O_5_, and 31% CaO).	(i) Enzymatic digestion (ii) Part of UC: not mentioned	*In vitro* study (1) Cell viability: (i) Scaffold had no significant inhibitory effect on MSC proliferation over time (ii) MSC proliferation gradually increased with incubation time	BM-MSCs, grown on BaG/Gel nanocomposite scaffolds, are possible sources for bone regeneration.
						*In vivo* study (1) Histological observations: (i) H&E staining: all MSC-seeded scaffolds successfully generated new bone and demonstrated an ongoing healing process at 4 and 12 weeks after transplantation. The UC-MSC-seeded scaffold showed significantly increased neovascularization compared to the others (ii) IHC staining: increased expression of OCN and ALP in the BM-MSC-seeded scaffold. Vascular endothelial growth factor (VEGF) expressed in all the groups of treated cell/scaffold. Increased neovascularization with the UC-MSC-seeded scaffold (iii) Histomorphometry: BM-MSC-seeded scaffolds showed more bone regeneration at 4 and 12 weeks	

10.	Todeschi et al. 2015 [81]	*In vivo*	(i) UC-MSCs (ii) BM-MSCs	(i) Ceramic scaffolds (Skelite; 4 × 4 × 4 mm cubes of 33% hydroxyapatite and 67% silicon-stabilized tricalcium phosphate, Si-TCP) (ii) Platelet-rich plasma (PRP) (iii) Conditioned medium (CM)	(i) Explant method (ii) Part of UC: not mentioned	(1) Histological assessment revealed the formation an immature bone-like structures and compact fibrous tissue in UC-MSC-seeded constructs (2) Polarized light examination revealed less organized collagen fibers in the UC-MSC-seeded scaffolds. The immature bone-like matrix in UC-MSC-seeded scaffolds was mostly filled with a loose connective tissue (3) Histological evaluation in an orthotopic mouse model showed that none of the bone defects had completely closed. However, gold MTC staining indicated the presence of red blood cells in blood vessel-like structures which is significant in the UC-MSC-transplanted group (4) Osteocytes were clearly detectable in the BMMSC-seeded scaffolds (5) However, human ALU sequences were not detected in osteocytes within the newly formed bone in the UC-MSC implants and nonseeded implants	UC-MSCs promote bone regeneration.

11.	Karadas et al. 2014 [74]	*In vitro*	(i) WJ-MSCs (ii) BM-MSCs (iii) Menstrual blood mesenchymal stem cells (MBMSCs)	(i) Collagen scaffolds with *in situ* calcium phosphate (CaP) (ii) Differentiation medium: (1) High-glucose DMEM (2) 10 nM dexamethasone (3) 50 *μ*g/mL ascorbic acid (4) 10 mM *β*-glycerophosphate (5) 10% FBS (6) 100 units/mL penicillin (7) 100 *μ*g/mL streptomycin	(i) Explant method (ii) Part of UC: not mentioned	(1) Cell proliferation assays: (i) WJ-MSCs on tissue culture polystyrene (TCPS) and collagen without CaP treatment increased proliferation (2) Cell attachment (fluorescence staining): (i) Good attachment of both cell types to the scaffold (ii) Confocal micrographs showed that the cells were able to penetrate into the pores (3) Osteogenic differentiation: (i) ALP assay: (a) WJ-MSCs showed better differentiation on untreated and CaP-containing foams than in growth medium (b) ALP levels were higher in WJ-MSCs grown on CaP-free foams than on TCPS (c) ALP activity was significantly higher in cells grown on collagen with CaP crystals formed in situ for both WJ-MSCs and MBMSCs (days 14 and 21)	Collagen foam with the use of CaP crystals formed *in situ* enhances the osteogenic induction of WJ-MSCs.
						von Kossa staining: (1) WJ-MSCs had higher ALP activity and denser mineral deposition compared to MBMSCs	

12.	Ramesh et al. 2014 [93]	*In vitro*	(i) WJ-MSCs	(i) Hydrogel alginate microspheres (ii) Osteogenic differentiation media: (1) Basal medium (2) 10 mM *β*-glycerophosphate (3) 1 mM dexamethasone (4) 5 mg/mL ascorbic acid	(i) Explant method (ii) Part of UC: not mentioned	(1) Characterization of osteodifferentiated WJ-MSCs via: (i) Bradford assay: calcium deposition increased in 2% alginate (ii) Alizarin Red staining: excellent matrix mineralization in WJ-MSCs immobilized on 2% alginate (iii) Immunocytochemical analysis (osteocalcin): significant expression of osteocalcin in WJ-MSC aggregates in 1.5% and 2% alginate at day 21 (2) Genotypic analysis of encapsulated WJ-MSCs showed that OCN and Runx2 were upregulated in 1.5% and 2% alginate	WJ-MSCs encapsulated in hydrogel alginate microspheres have osteogenic potential for stem cell-based tissue engineering.

13.	Baba et al. 2012 [16]	*In vivo* & *in vitro*	Human umbilical cord mesenchymal stem cells (hUC-MSCs)	Differentiation medium: (i) NH OsteoDiff Medium (ii) NH AdipoDiff Medium (iii) rhBMP2 (iv) Scaffold	(i) Enzymatic digestion (ii) Part of UC: not mentioned	(3) Osteogenic differentiation: strong calcium deposition Adipogenic differentiation: lipid droplet production (4) H&E staining: positive for bone tissue producing osteocalcin (OCN) (5) RT-PCR: high expression of RUNX2, ALP, and OCN as compared to undifferentiated cells	UC-MSCs supplemented with growth factors and serum have osteogenic differentiation potential for bone regeneration.

14.	Penolazzi et al. 2012 [89]	*In vitro*	(i) WJ-MSCs	(i) Porcine urinary bladder matrix (pUBM)	(i) Enzymatic digestion (ii) Part of UC: not mentioned	(1) Proliferation assays showed (i) pUBM did not have an effect on cells in suspension conditions but affected cells cultured in adherent conditions (ii) Viable cells were homogenously distributed over the entire scaffold (2) TUNEL assays showed (i) No apoptosis in hWJ-MSCs cultured in agarose-coated wells with increasing amounts of pUBM (ii) The scaffold upregulated cyclin D1 (iii) Matrix metanoproteinase (MMPI3) was lower but this did not affect *β*-catenin (3) Morphological characterization: (i) Scanning electron microscope (SEM) analysis showed (a) There was a significant interaction between the cells and the biomaterial (b) WJ-MSCs completely were enveloped in pUBM particles to form smooth spheroids, displaying an ECM network covering the surface (c) The cells and the biomaterial formed a dense structure (ii) X-ray energy-dispersive spectroscopy (EDX) showed that spheroids in osteogenic medium contained high amounts of calcium and phosphorous (high degree of mineralization) (iii) Transmission electron microscopy (TEM) showed the presence of focal contacts between cells and the pUBM scaffold (4) RT-PCR: (i) RUNX2 expression was not affected by pUBM, but increased upon osteoinduction (ii) WJ-MSCs seeded on pUBM were able to produce Col IAI and OPN after 21 days in osteogenic medium (iii) WJ-MSCs showed increased ALP activity and ability to deposit mineralized matrix (iv) OPN expression was higher in cells grown on pUBM scaffolds in osteogenic medium than in osteogenic medium alone	The combination of WJ-MSCs and pUBM shows the promise of scaffolds for bone regeneration.

15.	Wang et al. 2011 [94]	*In vitro*	(i) UC-MSCs	(i) Poly-L-lactic acid (PLLA) scaffold (ii) Osteogenic induction medium: (1) Low-glucose DMEM (2) 10% FBS (3) 1% penicillin/streptomycin (4) 100 nM dexamethasone, (5) 10 mM sodium *β*-glycerophosphate, (6) 50 *μ*g/mL ascorbic acid 2-phosphate (AA2P) (7) 10 nM 1*α*,25-dihydroxyvitamin D3 (i) Chondrogenic induction medium: (1) High-glucose DMEM (DMEM-HG) (2) 1% nonessential amino acids (NEAA) (3) 1x sodium pyruvate (4) 1x insulin-transferrin-selenium premix (ITS) (5) 50 *μ*g/mL (AA2P) (6) 40 *μ*g/mL L-proline (7) 100 nM dexamethasone (8) 10 ng/mL TGF-*β*1	(i) Enzymatic digestion (ii) Part of UC: not mentioned	(1) Biochemical assays were performed to assess (i) DNA content: osteogenic parts of the C-cell-O composites had higher DNA contents than the O-O composites and the osteogenic parts of the C-O composites (ii) Glycosaminoglycan (GAG) content: all osteogenic groups had similar GAG contents (iii) Hydroxyproline (HYP) content: osteogenic groups had a significantly higher HYP content (iv) Calcium content: osteogenic groups showed significantly increased calcium levels over time (2) Histological analyses: positive Alizarin Red staining in the osteogenic group (3) RT-PCR: (i) Collagen type II (ColII): not expressed in the osteogenic group (ii) Collagen type I (ColI): upregulated in all groups (iii) RUNX2: increased in the osteogenic group (iv) Aggrecan: increased significantly in chondrogenic group	WJ-MSCs are a suitable cell source for a sandwich approach strategy in osteochondral tissue engineering.

16.	Schneider et al. 2010 [85]	*In vitro*	Human mesenchymal stem cells (hMSC): (i) UC-MSCs (ii) BM-MSCs	(i) Scaffold: 3D collagen gel (ii) Osteogenic induction medium: (1) Low-glucose DMEM (2) 10% FCS (3) 100 nM dexamethasone (4) 10 mM sodium *β*-glycerophosphate (5) 0.05 mM/L-ascorbic acid 2-phosphate (iii) Adipogenic induction medium: (1) DMEM high glucose (2) 1 *μ*M dexamethasone (3) 0.2 mM indomethacin (4) 0.01 mg/mL insulin (5) 0.5 mM 3-isobutyl-1-methylxanthine (6) 10% FCS	(i) Enzymatic digestion (ii) Part of UC: not mentioned	(1) Scaffold: (i) 3D collagen gel underwent progressive contraction (ii) After osteogenic differentiation, collagen gels were stronger and harder with BM-MSCs and UC-MSCs (2) Characterization of BM-MSCs and UC-MSCs by (i) Osteogenic differentiation: UC-MSCs showed increased extracellular matrix (ECM) deposition by Alizarin Red (AR) staining (ii) Adipogenic differentiation: UC-MSCs showed a limited number of small lipid vacuoles stained by Oil Red O (ORO) (iii) Immunofluorescence: positive expression of collagen IV and laminin in UC-MSCs after 21 days of osteogenic differentiation on collagen gels (iv) Immunohistochemistry analyses: positive expression of osteopontin (OPN) and bisphosphonate [2-(2-pyridinyl) ethylidene-BP] (PEBP) in the collagen gel (3) TEM analysis: (i) Osteogenic differentiation: contraction of the collagenous matrix on the UC-MSC-seeded collagen surface (ii) Adipogenic differentiation: UC-MSC-derived lipid vacuoles were small and stained with toluidine blue (4) RT-PCR: UC-MSCs expressed collagen I, collagen III, collagen IV, and laminin (5) Cell migration: migrating UC-MSCs appeared as spindle-shaped cells with elongated cytoplasmic processes	UC-MSCs have a significant therapeutic impact in bone tissue engineering in the future.

17.	Hsieh et al. 2010 [14]	(i) *In vitro*	(i) WJ-MSCs (ii) BM-MSCs	Osteogenic differentiation medium: (1) DMEM (2) 10% FBS (3) 0.1 mM dexamethasone (4) 10 mM *β*-glycerophosphate (5) 50 mM ascorbic acid	(i) Enzymatic digestion (ii) Part of UC: not mentioned	(1) Array data showed that both BM-MSCs and WJ-MSCs expressed multilineage differentiation properties. (2) Real-time-PCR: BM-MSCs > WJ-MSCs in terms of (i) Adipocytic marker expression: *lipoprotein lipase* (LPL), leptin, peroxisome proliferator-activated receptor gamma (PPAR*γ*), and fatty acid-binding protein 4 (FABP4) (ii) Lipid accumulation (3) Osteogenic differentiation potential (i) BM-MSCs > WJ-MSCs when cultured in defined MesenCult (ii) Expressed higher levels of ALP, SPP-1, and RUNX2 (iii) BM-MSCs > WJ-MSCs when cultured in defined cultured in 10% FBS: (a) Expressed higher levels of ALP, osteopontin, and RUNX2 (4) ALP staining (better osteogenic ability)	WJ-MSCs are capable of differentiating into the osteogenic lineage, but BM-MSCs are superior.

18.	Hou et al. 2009 [15]	(i) *In vitro*	(i) hUC-MSCs (ii) BM-MSCs	(i) BMP-2 treatment: (a) Bone morphogenetic protein 2-blocking antibodies (BMP2 Ab) (b) Recombinant human BMP2 (rhBMP2) (c) Noggin (ii) Osteogenic differentiation medium: (1) DMEM/F12 (2) 10% FBS (3) Dexamethasone (4) Ascorbic acid (AsA) 2-phosphate (5) *β*-Glycerophosphate (iii) Adipogenic differentiation medium: (1) DMEM/F12 (2) 1% FBS (3) 100 nM dexamethasone (4) 1 nM insulin (iv) Chondrogenic differentiation medium: (1) DMEM/F12 (2) Dexamethasone (3) AsA (4) TGF-*β*1 (5) ITS^+^ Premix	(i) Enzymatic digestion (ii) Part of UC: not mentioned	(1) Trilineage differentiation (i) Osteogenesis: osteonectin, ALP, and RUNX2 were expressed (ii) Chondrogenesis: COL II, collagen type X (COL X), and aggrecan were expressed (iii) Adipogenesis: adipsin, PPAR*γ*, and lipoprotein lipase (2) ALP activity was significantly increased in BMP2-induced UC-MSCs (i) Western blot revealed activation of the BMP2 signaling pathway in both cell types via SMADs, p38, and extracellular regulated kinase activation	BMP2-induced UC-MSCs have good osteogenic differentiation (indicated by the activation of BMP2 signaling) and may be used in tissue-engineered bone.
